# Treatment of Necrotic Teeth Using Two Engine-Driven Systems and Patient’s Postoperative Pain: A Double-Blind Clinical Trial

**DOI:** 10.22037/iej.2016.3

**Published:** 2016

**Authors:** Vahid Zand, Amin Salem Milani, Ayla Hassani Dehkharghani, Mahdi Rahbar, Pardis Tehranchi

**Affiliations:** a *Department of Endodontics, Dental School, Tabriz University of Medical Science, Tabriz, Iran; *; b * Department of Operative and Esthetic Dentistry, Dental School, Tabriz University of Medical Science, Tabriz, Iran*

**Keywords:** Necrotic Teeth, Postoperative Pain, RaCe Files, Reciproc Files, Visual Analogue Scale

## Abstract

**Introduction:**

One of the most important reasons for postoperative pain is the extrusion of debris from the apical foramen during preparation and shaping of root canals. The aim of this clinical trial was to evaluate the severity of postoperative pain with the use of two different engine-driven NiTi systems.

**Methods and Materials:**

Ninety mandibular molars were randomly divided into two groups (*n*=45), and root canal cleaning and shaping was done using either RaCe or Reciproc instruments. The severity of postoperative pain was determined with visual analogue scale (VAS) at 4-, 12-, 24-, 48- and 72 h and 1-week intervals and postoperative pain was compared between the two groups. The chi-squared test and repeated-measures analysis were used to compare the data between the two groups.

**Results:**

Based on the results of the statistical analyses, the two groups were matched regarding the age and gender, with no significant differences. In addition, except for 4- and 24-h and 1-week intervals, postoperative pain was significantly less in the RaCe group compared to the Reciproc group (*P*<0.001).

**Conclusion:**

Based on the results of the present study, use of RaCe files for cleaning and shaping of root canals in necrotic mandibular molars resulted in less severe postoperative pain compared to Reciproc files.

## Introduction

Pain after root canal treatment is one of the most common complications in endodontics, with a prevalence rate of 2 to 20% [1]. The incidence of postoperative pain is reported to be 40% during the first 24 h and decreases over the time. In some cases the pain severity after treatment might exceed the preoperative levels, which is attributed to the exacerbation of inflammatory processes due to root canal debridement, especially in teeth with preexisting periradicular inflammation [2]. Other etiologic factors, have been suggested as well, including the remaining pulpal tissue, over instrumentation, traumatic occlusion and extrusion of medications, irrigation solutions, root canal debris and microorganisms from the apical foramen [3]. Of all the factors mentioned above, some preventable iatrogenic factors during root canal treatment (canal cleaning and shaping methods and the type of the files used, *i.e.* hand or rotary) are also held responsible for postoperative pain [4]. 

Despite the limitation of root canal treatment procedures to the apical end of the root canal, the extrusion of some root canal debris into the periapical area, do occur [5]. In spite of the various advantages of NiTi rotary systems, use of multiple files for increasing the size and achieving proper taper of the canal during preparation, results in an increased chair time [6]. With development of new NiTi rotary techniques, less apical extrusion of debris occur compared to hand files due to the Archimedes effect in association with copious root canal irrigation; therefore, there is less pain and discomfort after root canal treatment using rotary files [6]. 

In order to facilitate the root canal therapy procedure and decrease the chair time, some engine-driven single-instrument NiTi systems with the use of a new NiTi alloy, referred to as M-wire, are introduced that prepare the root canal system with a reciprocal back-and-forward motion with a speed of 300 rpm (150 degrees counterclockwise and then 30 degrees clockwise). The Reciproc instruments (VDW, Munich, Germany) available at three different sizes and tapers; R25 (25/0.08), R40 (40/0.06) and R50 (50/0.05) [7-9]. In reciprocation movement, the instrument is driven first in a cutting direction and then reverses to release the instrument. One complete rotation of 360^°^ is completed in several reciprocating movements. The reciprocating movement relieves stress on the instrument and, therefore, reduces the risk of cyclic fatigue. At the same time, reciprocation ensures that the instrument stays centered in the canal [10].

The advantage of reciprocating movements over rotary movements is that there are lower risks of file fracture due to the continuous rotation at canal curvature areas; however, these movements have some disadvantages such as limited cutting efficacy, the need for the application of more force toward the root apex and lower ability to remove debris from the root canal [11]. In root canal preparation techniques the control over the instrument and prevention of the extrusion of debris from the apical foramen are the main factors for decreasing the incidence and severity of postoperative pain; therefore, it might be possible to exert more control over the above factors in favor of decreasing the postoperative pain with the use of new root canal preparation systems such as the single-file systems, including Reciproc [12]. Currently, emphasis is placed on the shortest time possible for root canal preparation. In addition, since rotary files can be sterilized, it is possible for cross-contamination between patients due to the residual debris on the files [13]. Therefore, the present clinical trial was designed to determine the severity of postoperative pain after using the single-file root canal preparation system with Reciproc system in comparison to preparation with RaCe system using the crown-down technique in necrotic teeth. The results of this study might help choose an appropriate technique for root canal treatment with less postoperative complications and discomfort.

## Materials and Methods

In this double-blind clinical trial, the frequency and severity of postoperative endodontic pain were evaluated and compared between two groups of subjects who underwent root canal treatment with RaCe (FKG Dentaire, La-Chaux-de Fonds, Switzerland) and Reciproc (VDW, Munich, Germany) systems. The protocol of the study was approved by the Ethics Committee of Tabriz University of Medical Sciences (Grant No.: TBZMED.REC.1394.659). A pilot study was carried out with 5 samples to determine the samples size because no similar study was available when the research plan was proposed. Considering *α*=0.05, study power of 80% and an acceptable level of difference in pain severity, the final sample size was estimated to be 90 samples (*n*=45). The study was carried out on patients referring to the Department of Endodontics, Tabriz Faculty of Dentistry during one year (from November 2013 to November 2015). All the patients who were eligible to be included in the study signed an informed consent form. 

The inclusion criteria consisted of age over 18 years, systemic health, presence of mandibular molars with necrotic pulp, absence of pain before treatment, normal periapical status or radiographic lesions under 2 mm in size, tooth sensitivity to percussion, the capacity of tooth restorability, absence of a sinus tract and absence of periapical abscess or facial cellulitis. The exclusion criteria consisted of systemic diseases, breastfeeding, pregnancy, allergy to lidocaine, healthy pulp, reversible pulpitis, irreversible pulpitis, periapical lesion measuring over 2 mm, absence of lip anesthesia after administration of local anesthesia, pulpal bleeding after pulp exposure, use of analgesics 48-72 h before initiation of treatment, use of corticosteroids one week before treatment, sensitivity to palpation and age under 18 years. 

Clinical diagnosis of necrotic pulp was established by the absence of response to thermal and electric tests. The pulpal status of each tooth was evaluated with thermal tests consisting of cold test with Green Endo Ice (Hygenic Corp, Akron, OH, USA) and heat test with hot gutta-percha and electric pulp tester (The Element Diagnostic Unit, Sybron Endo, Glendora, CA, USA); the periradicular status was evaluated with percussion, palpation and preoperative radiographies. Local anesthesia was achieved with inferior alveolar nerve block injection of 2% lidocaine containing 1:80000 epinephrine (Darupakhsh, Tehran, Iran). After 15 min, the subjects were questioned about the presence of lip numbness. In some cases supplementary injections were used; followed by isolation of teeth with a rubber dam and endodontic access cavity preparation. Then the canal orifices were located and a rubber dam was used for isolation. The root canal path and length were determined with a #15 K-file (Dentsply Maillefer, Ballaigues, Switzerland) and the working length was determined using an apex locator (Root ZX, J. Morita USA, Inc., Irvine, CA, USA). Then the WL was confirmed with digital radiography using Kodak RVG.

The subjects were randomly divided into two groups; in group A, RaCe rotary files (FKG Dentaire, La-Chaux-de Fonds, Switzerland) and in group B Reciproc files (VDW, Munich, Germany) were used. RaCe instruments were used in crown-down technique according to the manufacturer’s instructions; with the following sequence: 40/0.10 and 35/0.08 for the preparation of the coronal third of each root canal followed by 30/0.06 in the middle third, 25/0.04 in the apical third and 30/0.04 up to the working length. The final apical size was achieved with 30/0.04 or 35/0.04 file. Reciproc instruments were also used according to the manufacturer’s instructions; R25 (25/0.08) was used for narrow canals and R40 (40/0.06) was used for wide canals.

Concomitant with the use of files for cleaning and shaping of the root canals, 17% ethylenediaminetetraacetic acid (EDTA) (Ariadent, Tehran, Iran) in gel-form was used as a lubricant. During all the preparation procedures with both systems, the root canals were irrigated with 30 mL of normal saline using a syringe connected to a 25-guage needle after each file. The needle was inserted into each root canal as far as possible, without binding. Finally the pulp chamber and the root canals were irrigated with 5 mL of 2.5% sodium hypochlorite solution. After the final rinse with normal saline solution, the root canals were dried with paper points and the standard ISO-sized matching master cones were fitted and checked with radiography. Then the root canals were obturated with gutta-percha (Meta Biomed, Cheongju, Korea) and AH-26 sealer (Dentsply, Tulsa Dental, Tulsa, OK, USA), using the lateral compaction technique. A temporary filling material (Zoliran; Golchai, Tehran, Iran) was placed and the occlusion was checked. Then the patient was referred to the restorative department for the final restoration.

Visual analogue scale (VAS) was explained to all the patients orally and in written form so that the patients would be able to mark their pain severity at 4-, 12-, 24-, 48- and 72-h and 1-week postoperative intervals. 

The following VAS classification was used [14]: 0; no pain, 0‒20; mild pain, 21-40; moderate pain, 41-60; severe pain, 61-80; very severe pain and 81-100; the most severe pain conceivable. 

It should be pointed out that the patients were not aware of the technique used and the forms were finally analyzed by a blinded clinician. The Statistical Package for Social Science software (SPSS, version 21.0, Chicago, IL, USA) was used for statistical analysis of data. The T-test was used to compare quantitative data between the two groups; the chi-squared test was used to compare qualitative data between the two groups. Repeated-measures analysis was used to compare pain severities at different time intervals within each group and between the two groups. Statistical significance was set at 0.05.

## Results

**Figure 1:**
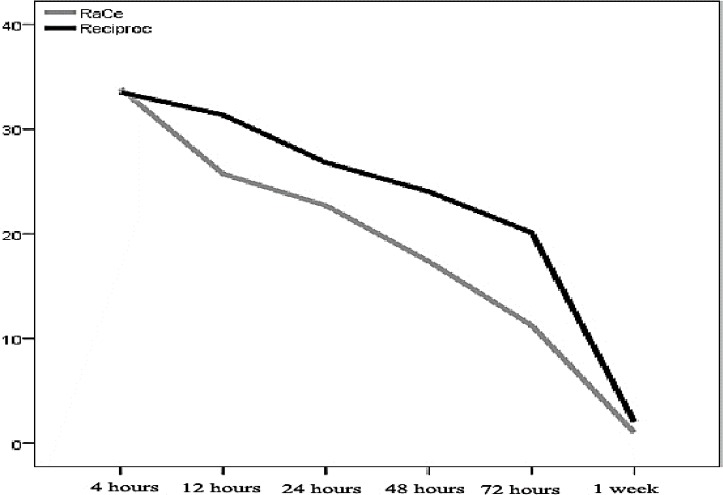
Mean changes in pain severity based on visual analog scale (VAS) in the two groups at different time intervals

Table 1 summarizes and compares the demographic data of the subjects. Based on data presented in Table 1, the two groups were matched in terms of age and gender, with no significant differences. Table 2 presents the means of pain severities on VAS at different intervals in both groups; Figure 1 shows these means separately at each evaluation intervals. 

**Figure 2:**
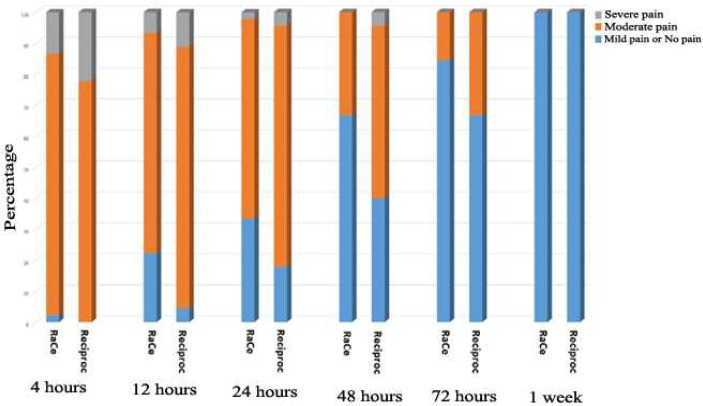
The frequency of pain quality in two groups at different time intervals

Base on repeated-measures analysis, in both groups the severity of pain decreased significantly from 4 h postoperative interval to 1 week except for the 24-h interval (*P*<0.001). Based on the results of the same test and also Figure 1, the severity of pain in the RaCe group was significantly lower at all the intervals except for 4-h and 1-week intervals (*P*<0.001). Table 3 presents the frequencies of pain qualities in the two study groups at different time intervals and Figure 2 presents the percentages of these pain severities. Based on the results of the chi-squared test, there were no significant differences at 4- and 24-h and 1-week postoperative intervals; however, the frequency of pain-free and low pain statuses at 12-, 48- and 72-h intervals were significantly higher in the RaCe group.

## Discussion

It is important to prevent pain and inflammation after endodontic treatment. Although very severe post endodontic pain is uncommon, still a notable number of patients complain of mild, moderate and severe pain after endodontic procedures. Although a number of factors, including irreversible pulpitis, preoperative pain and teeth with large periapical lesions, have been reported as predictors of postoperative endodontic pain, it is still possible to avoid it by modifying the root canal therapy technique or at least decrease its severity. One of the most effective techniques to prevent such pain is providing an effective treatment by creating properly cleaned and shaped root canals so that the odds of apical extrusion of root canal contents is minimized. NiTi rotary instruments have become very popular in recent years because they facilitate root canal shaping and decrease iatrogenic errors at the same time; in addition, they are more flexible than manual stainless steel instruments [15-17].

Furthermore, the crown-down preparation technique is a very useful due to decreasing stresses on the rotating instruments and since coronal widening of the root canal facilitates the penetration of the disinfecting solutions into the apical third of the canal,

this technique improves root canal debridement [18, 19]. During endodontic procedures on extracted teeth, it is common to observe the formation of “endodontic worm”, which refers to a tubular mass of root canal debris produced by extrusion through the apical foramen during the procedure, improper irrigation and lack of recapitulation. In the clinic, this residual debris is the main etiologic factor for postoperative pain. This special feature consists of bacteria, tooth fragments, irritants and inflamed or necrotic pulp that are all considered components of the root canal contents. When such debris enters the apical tissues, it becomes toxic and gives rise to postoperative pain and inflammation [20]. 

**Table 1.** The demographic data of subjects in the two study groups

**Variable**		**RaCe group (** ***n*** **=45)**	**Reciproc group (** ***n*** **=45)**	***P*** **-value**
**Age [Mean±SD (Max-Min)]**		33.22±8.97 (19‒58)^*^	33.73±10.35 (19‒59)	0.80
**Gender**	**Male N (%)**	18 (40)	23 (51.1)	0.29
	**Female N (%)**	27 (60)	22 (48.9)	

**Table 2:** The mean pain severity based on VAS in the two study groups at different time intervals

**Group/Time interval**	**4 h**	**12 h**	**24 h**	**48 h**	**72 h**	**1 week**
**RaCe**	32.89±5.01	25.71±4.31	22.69±4.12	17.36±5.41	11.24±3.24	1.00±8.97
**Reciproc**	33.51±8.46	31.36±6.04	26.80±8.29	24.02±6.68	20.11±7.19	2.04±1.24

In the present study, single-file reciprocating preparation technique with the Reciproc system resulted in more severe postoperative pain compared to the RaCe system with the crown-down technique and full rotational movement. However, the differences between 4-h and 1-week postoperative intervals were not significant statistically. Since the possible confounding factors such as patients’ age and gender, the tooth type, the type of the background pathologic conditions, the type of the irrigation solution used and the dentist rendering treatment, were similar in both groups, the difference in the severity of postoperative pain can be attributed to the technique/system used for root canal preparation [20]. 

To date, few studies have been carried out on this subject. Gambarini *et al.* [20] compared the incidence and severity of postoperative pain after root canal treatment of necrotic teeth with RaCe rotary files and the single-file Reciproc system. The results showed higher incidence and more severity of postoperative pain in the Reciproc group, which was statistically significant. Finally, it was concluded that in cases of pulp necrosis, NiTi rotary crown-down technique was superior. The results of the present study, was consistent with the study above. However, the present study had more samples size and only mandibular molars were evaluated. In the aforementioned study maxillary and mandibular molars and premolars were evaluated, which are very different and cannot be compared in terms of their innervation and anatomic features.

In a recent study by Pasquilini *et al.* [21], the patients’ quality of life after treatment (including pain) was compared in two groups of patients undergoing treatment with the rotary technique (ProTaper) and reciprocating system (WaveOne). Pain was significantly less in the rotary group. However, the sample size was smaller than that in the present study. 

According to Gambarini *et al.* [22], two possible mechanisms are involved in higher incidence of extrusion of debris and more severe pain with the use of the reciprocating systems: In the reciprocating technique, the reciprocating movements occur at a wider cutting angle and at a smaller liberating angle. A small liberating angle increases the possibility of pushing root canal debris towards the apex. Moreover, it has been demonstrated that NiTi rotary instruments have the best performance with the crown-down technique because the coronal and apical thirds are cleaned at the beginning and end, respectively [23]. This reduces the odds of extrusion because the coronal third of the canal is cleaned before the apical third. In addition, instruments are inserted slowly, with more care and in a passive manner.

On the other hand, the Reciproc technique uses a single file with an increased taper, which is directly inserted towards the apex. In the majority of cases, the reciprocating file should be moved to the apex with force in order to reach the working length, which increases the odds of root canal debris being pushed toward the apical foramen. In addition, reciprocating files have lower cutting efficacy compared to rotary files, which increases the frictional force and the need for rotation due to the entanglement of the residual debris within dentin [20]. 

**Table 3:** The frequencies of pain qualities in the two groups at different time intervals

**Time interval**	**Pain quality**	**RaCe group**	**Reciproc group**	***P*** **-value**
**48 h**	Non or mild	0	1	
	Moderate	35	38	0.35
	Severe	10	36	
**12 h**	Non or mild	2	10	
	Moderate	38	32	0.04
	Severe	5	3	
**24 h**	Non or mild	8	15	
	Moderate	35	29	0.22
	Severe	2	1	
**48 h**	Non or mild	18	30	
	Moderate	25	15	0.02
	Severe	2	1	
**72 h**	Non or mild	30	38	
	Moderate	15	7	0.05
	Severe	0	0	
**1 week**	Non or mild	45	45	
	Moderate	0	0	-
	Severe	0	0	

In addition to the tendency to push the intracanal debris beyond the apex, other factors too, might be involved in increasing pain severity in such patients, including the effect of preoperative pain and the pulpal pathology [24]. For example, it has been shown that severe postoperative pain and flare-up in patients with pulpal necrosis are more common than in patients with vital pulps [20, 25-27].

In addition, attention should be paid to differences in classification of pain severity and also differences in evaluated patients [28-31]. For instance, it has been demonstrated that women exaggerate postoperative pain severity compared to men [32] and such gender difference in the patients evaluated in different studies might result in differences in the reported incidence and severity of pain. 

Another important consideration in the present study was the absence of significant differences between the two groups at 4-h and 1-week postoperative intervals. The absence of differences between the two groups shortly after the treatment procedure might be attributed to the effect of local anesthetic agents. On the other hand, as mentioned previously, the principal mechanism of pain in these patients is the extrusion of root canal debris into the surrounding tissues and induction of inflammation and infection, and it appears that 4 h does not provide the adequate opportunity for the appearance of such pain was reported in all the patients in both groups that might confirm the possible mechanisms discussed previously [33]. Consistent with this finding, some studies, have shown that the frequency of pain after endodontic treatment is usually low after 4 days, irrespective of the technique used [4, 34, 35].

This issue draws more attention to the fact that some studies have reported similar success rates for the rotary and reciprocating techniques in eliminating infection. For example, in two studies by Martinho *et al.* [36, 37], the two rotary and reciprocating techniques were equally successful in eliminating infection as determined by the amount of residual endotoxins and cultivable bacterial counts. Therefore, the role of the added inflammation becomes more noticeable at postoperative intervals.

## Conclusion

Based on the results of the present study; preparing and shaping the root canals in the necrotic mandibular molars with RaCe Rotary systems, resulted in less severe postoperative pain compared to the Reciproc files.
